# Characterization of D-17 Canine Osteosarcoma Cell Line and Evaluation of Its Ability to Response to Infective Stressor Used as Alternative Anticancer Therapy

**DOI:** 10.3390/ani10111981

**Published:** 2020-10-28

**Authors:** Paola Modesto, Jordi Leonardo Castrillo Fernandez, Isabella Martini, Roberto Zoccola, Maria Concetta Pugliano, Chiara Grazia De Ciucis, Maria Goria, Angelo Ferrari, Elisabetta Razzuoli

**Affiliations:** 1 National Reference Center for Veterinary and Comparative Oncology (CEROVEC), Piazza Borgo Pila 39/24, 16129 Genoa, Italy; leonardocastrillo93@gmail.com (J.L.C.F.); isabella.martini@izsto.it (I.M.); mariaconcetta.pugliano@izsto.it (M.C.P.); cdeciucis@yahoo.it (C.G.D.C.); angelo.ferrari@izsto.it (A.F.); 2 Istituto Zooprofilattico Sperimentale del Piemonte, Liguria e Valle D’Aosta, Via Bologna 148, 10154 Turin, Italy; roberto.zoccola@izsto.it (R.Z.); maria.goria@izsto.it (M.G.)

**Keywords:** D-17, osteosarcoma, cytokines, gene expression, innate immunity, *Salmonella typhimurium*

## Abstract

**Simple Summary:**

Osteosarcoma (OSA) is the most common primary bone tumor both in dogs and in humans. Canine and human OSA share common characteristics making dogs a good model in comparative oncology. In the last years, in order to reduce animal testing, researchers shifted their attention to in vitro studies using cell lines. Aim of this work is to understand if cells obtained from canine metastatic pulmonary OSA can be a good model for cancer studies, both in humans and dogs. Results of this study were obtained by: the characterization of the expression of genes involved in the innate immune response, the sequencing of a single gene with a key role in immune response and the evaluation of the capacity of these cells to interact with microorganisms that can be used as alternative anticancer therapies. Obtained data were in agreement with those reported in literature regarding the expression of genes both in spontaneous tumors and in vitro cell lines. So, they confirmed the maintenance of cell line D-17 of the pulmonary metastatic OSA characteristics. The selected cells also demonstrated the ability to interact with the microorganism, this suggests that they may be a possible model for the preliminary evaluation of new therapeutic approaches based on the use of bacteria.

**Abstract:**

Osteosarcoma (OSA) is a rare cancer both in human and dog although the incidence rate in dogs is 27 times higher than in human. Many studies employed D-17 as cell line for in vitro test to evaluate conventional anticancer therapies; however, little is known about D-17 cell line. The aim of our study was to evaluate the basal level of gene expression of pivotal molecules in the innate immune response and cell cycle regulation and to establish the ability of this cell line to react to *Salmonella typhimurium (ST)* infective stressor. *IL15*, *IL10*, *iNOS*, *TLR5*, *CD14*, *PTEN* and *IL18* were expressed in an inconsistent manner among experiments. The other genes under study were expressed in all samples. *ST* showed ability to penetrate D-17 causing pro-inflammatory response. Our results outline the expression in D-17 of important genes involved in innate immune response. These results provide important data on D-17 basal gene expression profile useful for in vitro preliminary evaluation of new therapeutic approaches.

## 1. Introduction

Osteosarcoma (OSA) is a tumor arising from mesenchymal stem cells that can differentiate in fibrous tissue, cartilage, bone and can have chondroblast, fibroblastic, and osteoblastic components [1,2]. OSA is the most common primary tumor in the bone both in dogs and in humans; although a rare disease in both species [3]. However, OSA incidence rates in dogs are about 27 times higher than in humans [4]. The higher incidence rate of canine OSA makes dog a good model for human disease [5]. OSA can affect appendicular or axial skeleton; nevertheless, it is most frequently found in the long bones of the extremities both in humans (up 90% of cases) and dogs (estimated up of 80% of cases) while in other species occurs most commonly in axial skeleton or flat bone [3,4]. Dogs are exposed to many of the same environmental factors as humans, as they share the same living environment. Similar risk factors, including sex and environment, lead to tumor development and progression in dogs and people. Prognosis is relatively poor, in humans OSA survival rates is 5 years not having improved in decades. For dogs, we have one-year survival rates around ~45%. It is obvious that improved and novel treatment regimens are urgently required in both humans and dogs to improve survival in subject with OSA [6]. The ability to rapidly advance therapeutics in OSA is limited by the low incidence of this disease in humans. OSA is more prevalent in dogs; this can provide a significantly larger patient population in which to evaluate new treatment strategies. For these reason recent studies proposed the dog as a study model in comparative oncology [4]. However, the need to reduce animal testing has shifted the attention of the scientific community to cell cultures; in particular, the canine OSA cell line D-17 is often used during in vitro studies [7]. Nevertheless, this line has not been described in terms of basal gene expression and interaction with infectious stressors. This is an important topic because even if the possibility to use infectious stressors like bacteria as anticancer therapy has been known from decades, its feasibility is still new, and some recent studies investigated this topic [8]. In this conceptual framework, the aim of our study was: (1) to characterize the D-17 basal expression of the genes involved in the innate immune response and in the regulation of the cell cycle, also evaluating the effect of cellular passages on these parameters; (2) to characterize (C-X-C motif) chemokine receptor 4 CXCR4 exclusive receptor for cell-derived stromal factor1 (SDF-1) expressed by cancer and many immune cells and involved in the regulation of immune response; (3) to evaluate the ability of the cell line to interact with one infectious stressor, already suggested as an alternative anticancer therapy.

## 2. Materials and Methods

### 2.1. Cell Culture

D-17 cells are cell line isolated in 1969 from a 11 years old female dog with metastatic OSA in lung (https://www.microbiologyresearch.org/docserver/fulltext/jgv/25/1/JV0250010021.pdf?expires=1600679081&id=id&accname=guest&checksum=07FB36A29896DC291EB7D182C1A566C1). We purchased the cell line from IZSLER biobank OIE (http://www.ibvr.org/) at 252th passage and used the cell line for different experiments. Among the different passages (255th, 262nd and 305th) the cells were conserved at −80 °C and then thawed and grown in a mixture of Eagle’s Minimum Essential Medium in Earle’s (MEM, Carlo Erba Reagents s.r.l., Milan, Italy) enriched with L-glutamine 4 mM (Carlo Erba Reagents), 10% (v/v) Fetal Bovine Serum (FBS, GIBCO™, Thermofisher Scientific, Milan, Italy) and a mixture of Antibiotics (penicillin and streptomycin, 1% v/v, Carlo Erba Reagents). After each passage cells morphology was checked by microscopy.

Cells were seeded into 12-well tissue culture plates (2 mL per well, 2.5 × 10^5^ cells/mL) and incubated at 37 °C in 5% CO_2_ until confluence (16–24 h). In order to evaluate basal gene expression, cells were tested at 255th, 262nd and 305th passages; when cell reached confluence were incubated for 24 h to evaluate the effects of monolayer aging. Each experiment was repeated six times.

### 2.2. Reference Genes Selection

In order to select suitable reference genes for cell line D-17, a panel of candidate genes was selected from those reported in the literature to be able to amplify mRNA alone [9,10,11]. For the reference genes *RPS19* and *SDHA*, new primers were designed on exon-exon junctions, based on the *Canis lupus familiaris* sequences deposited in ESEMBL database (https://www.ensembl.org) using Primer3 software version 0.4.0 (https://bioinfo.ut.ee/primer3-0.4.0). Again, the primer pairs were tested to verify the amplification of mRNA alone. Primer pairs consisting of the forward primer from the literature and the designed reverse primers were the best assays and were used for the subsequent analyses (Table 1).

Total RNA was extracted from 10^6^ cells using RNeasy Mini Kit (Qiagen s.r.l., Milan Italy) through the Qiacube System (Qiagen s.r.l., Milan, Italy) in accordance with the manufacturer’s instructions. Retro-transcription reaction was carried out using OneScript^®^ cDNA Syntesis Kit (Applied Biological Materials Inc. Richmond, BC, Canada) with random primers and 30 μg of total RNA according to the manufacturer’s instructions. Controls were added to retro-transcription run: a negative sample without template and a sample containing RNA extract without retro-transcriptase.

Real Time qPCR was performed on CFX96™ Real-Time System (Bio-Rad, Milan, Italy) using Power SYBR^®^ Green PCR Master Mix kit (Applied Biosystems, Milan, Italy) adding a negative control to each run and a genomic DNA sample. The amplification reaction mix was constituted by: 1X Power SYBR^®^ Green PCR Master Mix kit, 50 nM of each primer (Carlo Erba Reagents, Cornaredo Italy) and 2 µL of cDNA, in a total volume of 20 µL. PCR thermal profile was: 95 °C for 10 min, and 40 cycles of 95 °C for 15 s; 60 °C 2 min. In order to verify the ability of each primer pair to detect only mRNA a step for the analysis of the melting curve has been added to the amplification thermal profile. All the run was performed on CFX96™ Real-Time System (Bio-Rad, Milan, Italy). For the evaluation of the efficiency (E) and correlation coefficient (R^2^) of the different assays, standard curves were performed using a 1:5 scalar dilutions of a pool of cDNA; each dilution was tested in triplicate. Each D-17 set samples was analysed with each assay according to the aforementioned protocol. The raw cycle-threshold (Cq) data of the RT-qPCR have been transformed into quantitative data (Q) with the formula Q = E ^(Min Cq - Sample Cq)^, where Min Cq is the lowest Cq detected for each gene in set of samples. The RT-qPCR data were analysed to verify the stability of the expression of the candidate reference genes using the NormFinder software version 0.953 [12] based on the recommendations of the developers and using the default settings.

### 2.3. Gene Expression Analysis

In this study, we evaluated the expression of the following genes: *ErbB2*, *TGFβ*, *CD44*, *IL6*, *IL10*, *IL15*, *IL8*, *CD14*, *NF-kB/p65 (p65)*, *IL18*, *TLR4*, *TLR5*, *TP53*, *MD2*, *MyD88*, *STAT5*, *iNOS*, *CXCR4*, *RAD51* and *PTEN* [13,14,15,16,17,18,19,20,21,22,23,24,25]. Genes selection has been made on the basis of their pivotal role in immune response to bacteria (*CD44*, *IL6*, *IL8*, *CD14*, *p65*, *IL18*, *TLR4*, *TLR5*, *MD2*, *MyD88*, *STAT5*, *iNOS*,) [26] or OSA development and metastasis (*ErbB2, TGFβ, CD44, TP53, MD2, MyD88, STAT5, CXCR4, RAD51, IL8, IL6* and *PTEN*) [27,28,29,30,31,32].

Total RNA was extracted from 10^6^ cells with vitality of 90% ± 94% of using RNeasy Mini Kit (Qiagen s.r.l., Milan Italy) by the Qiacube System (Qiagen s.r.l., Milan, Italy) in accordance with the manufacturer’s instructions. RNA concentration was tested by Qubit 3.0 Fluorometer and RNA quality by bio-photometer. Retro-transcription reaction was carried out using OneScript^®^ cDNA Syntesis Kit (Applied Biological Materials Inc. Richmond, BC, Canada) with random hexamers according to the manufacturer’s instructions.

Each sample was reverse-transcribed using 50 ng of total RNA Controls were added to retro-transcription run: a negative sample without template and a sample containing RNA extract without retro-transcriptase. Real-Time PCR amplification was performed on CFX96™ Real-Time System (Bio-Rad, Milan, Italy). We used primer set in use in our laboratory (Table 2). The expression of the above genes was assessed by RT-qPCR using Syber Green chemistry (Bio-Rad, Milan, Italy). PCR reaction had a final concentration of 0.2 µM of each primer; 1X of Sybr Green and 2 μL of template in a total volume of 20 μL. A negative control was added to each run. The used thermal profiles have been previously described by Razzuoli et al. [33]. All the run was performed on CFX96™ Real-Time System (Bio-Rad, Milan, Italy).

To evaluate the basal level of gene expression, a cut-off for PCR-negative samples was set at Cq 39, so that the positive ones showed Cq values < 39. The relative expression of the selected genes was calculated using the formula 2^-ΔΔCq^.

### 2.4. Immunocytochemical Assay

Immunocytochemical analysis were performed using the following antibodies: polyclonal Rabbit CXCR4 (Sigma Aldrich, Saint Louis, MO, USA) and Vimentin (VIM, Clone V9, Dako Denmark A/S, Glostrup, Denmark). Cell untreated with primary antibody were used as negative control; moreover, continuous cell line obtained from mammary cancer was tested as positive control. The dilutions were performed with 1% Bovine Serum Albumin (BSA, Applichem GmbH, Darmstadt, Germany) in Phosphate Buffered Saline (PBS, AMRESCO, VWR Int., Milan, Italy). D-17 cells (2.5 × 10^5^ cells/mL) were seeded into sterile Petri dish with a slide inside and incubated at 37 °C in 5% CO_2_ for 24 h, fixed in methanol (Carlo Erba Reagents, Cornaredo, Italy) at 4 °C for 10 min and air-dried. After this step, D-17 were washed twice for 5 min with PBS and treated with 8% BSA in PBS for 45 min at room temperature. Cells were treated with the primary antibody diluted as follow: VIM 1:200 for 30 min and CXCR4 1:500 overnight. Negative controls were treated with the same protocol and 1% of BSA. Afterward, samples were washed three times for 5 min with PBS and treated with the secondary antibody (Dako EnVision^+^ Dual Link System-HRP, Dako North America): CXCR4 for 50 min and VIM for 30 min. Then, cells were washed three times for 5 min with PBS and the color has been developed with 3,3′-diaminobenzidine (DAB, Dako Liquid DAB+ Substrate Chromogen System For Autostainer, Dako North America): VIM for 2 min and CXCR4 for 1 min; the reaction was blocked in distilled water for 5 min. Finally, the slides were colored with hematoxylin (Sigma Aldrich) and observed by optical microscope.

### 2.5. Innate Immune Response to Infective Stressor

To evaluate the ability of D-17 to respond to invective stressor we used the *Salmonella typhimurium* (ST) model in agreement with previous study [26]. Briefly, *ST* in mid-log phase cultures was re-suspended at 10^8^ CFU/mL. D-17 cells were treated with 1 mL of bacterial suspension (MOI 100 ufc/cells) for 1 h at 37 °C in 5% CO_2_ and used for three experiments: (1) Invasivity, (2) Cells vitality, (3) Immunomodulation.

#### 2.5.1. Experiment 1: Bacterial Invasion

After removal of bacteria, cells were washed three times with medium only and treated with PBS, containing 300 μg/mL colistin sulphate (Microbiol & C. s.n.c., Cagliari, Italy) at 37 °C in 5% CO_2_ for 2 h to remove all extracellular bacteria. Cells were lysed with 200 µL of 1% Triton X-100 (Sigma Aldrich, Milan, Italy) in PBS at room temperature for 5 min then, 900 µL of PBS were added to each well; the resulting cell suspension was vortexed, serially diluted and seeded on Xylose Lysine Deoxycholate (XLD, Sigma Aldrich, Milan, Italy). After 24–48 h of incubation at 37 °C *ST* were counted. Cells treated with medium only were used as negative control. The experiment was performed twice.

#### 2.5.2. Experiment 2: Cell Viability after Treatment

After 1 h of *ST* exposure (see Section 2.3) cells vitality was checked using the LUNA II Automated Cell Counter (LUNA^TM^ Logos Biosystems, Inc., Anyang, Korea). Cells were detached with trypsin (Sigma Aldrich, Milan, Italy) and blocked with complete medium; then, 10 μL of Trypan Blue 0.4% (Logos Biosystems, Inc., Korea) were added to 10 μL of cells suspension to evaluate cell viability. Cells treated with medium only were used as untreated control. The experiment was performed twice.

#### 2.5.3. Experiment 3: Modulation of Innate Immune Responses

The modulation of gene expression was evaluated after D-17 treatment with *ST* (see Section 2.3) and incubation at 37 °C in 5% CO_2_ for 1 h. Then, cells were washed three times with medium only and again incubated at 37 °C in 5% CO_2_ for 3 h with fresh completed medium. As negative control cells treated with medium only were used. The experiment was repeated three times.

### 2.6. Sequencing of the Key Gene CXCR4

In order to evaluate the presence of mutations that could modify CXCR4 activity, we compared sequences obtained in cancer cell line with those of animal healthy tissues, sequencing was performed on D-17 and 5 non-neoplastic canine tissues (2 lymphonodes, 2 kidneys and 1 liver). D-17 were tested at the 302th and 255th passages and each experiment was repeated three times. Genomic DNA was extracted from 1 × 10^6^ cells or from 25 mg of tissue using QIAmp DNA Mini kit (Qiagen, Milan, Italy) in accordance with the manufacturer’s instructions. Tissue/cells rupture occurred with mechanical cut followed by incubation with proteinase K addition of absolute ethanol allowed the formation of bonds between extracted DNA and resins present in columns supplied by kit, DNA was cleaned of contaminants by subsequent washing with alcohol-based solutions and finally eluted in 100 μL of Tris-EDTA buffer (TE, Sigma Aldrich, Milan, Italy). In domestic dogs (*Canis lupus familiaris*) *CXCR4* gene is located within chromosome 19 [NC_006601.3 (38874650.38877740, complement)] and is involved in the transduction for the synthesis of a 1129 bp mRNA (GenBank: DQ182699.1). During the first stages of the study, the characterization of the *CXCR4* gene has been obtained using primers described by Fan and collaborators [33]. These primers amplified a 364 bp portion of the gene (from 500 to 864 nucleotide of the reference) not allowing us to highlight possible mutations located elsewhere. In order to increase the possibility of detecting mutations, specific primers have been designed on the reference sequence to target a 902 bp segment of a conserved encoding region (Table 3) using Primer3 software version 0.4.0 (https://bioinfo.ut.ee/primer3-0.4.0). PCR reaction mix contained: 1X PCR Buffer, 1 mM MgCl_2_, 1.25 U hot start Taq polymerase, dNTPs mix 10 mM each, 0.25 µL of each primer (Carlo Erba Reagents, Cornaredo, Italy). Thermal profile was 94 °C for 15 min, 35 cycles of 94 °C for 30 s, 60 °C for 30 s and 72 °C for 60 s, a final step at 72 °C for 10 min was added. A negative control was added to each run. The PCR run were performed on GeneAmp9700 (Applied Biosystems, Monza, Italy). After electrophoresis in agarose gel, amplification products were purified using High Pure PCR Product Purification Kit (Roche diagnostics, Monza, Italy) and primers producing overlapping sequences (see Table 3) were used to obtain the consensus sequence of the entire 902 bp segment. Sequencing reaction products were purified using the DyeEx 2.0 Spin Kit (Qiagen, Milan, Italy) for removal of unincorporated dye terminators. Capillary electrophoresis was performed using Applied Biosystems 3500 genetic Analyzer (Thermo scientific, Monza, Italy) and obtained sequences were aligned with BioEdit Sequence Alignment Editor version 7.2.5. Forward and reverse sequences were aligned with reference sequences using the ClustalW multiple alignment function [34].

### 2.7. Statistical Analyses

Gene expression data were submitted to a Kolmogorov-Smirnov test to check Gaussian distributions; significant differences within normal distributions were checked by one-way ANOVA (gene expression) or Student’s *t* test (effect of aging on gene expression). The significance threshold was set at *p* < 0.05 (Prism 5, GraphPad Software).

## 3. Results

### 3.1. Reference Gene Selection and Basal Gene Expression

**Figure 1:**
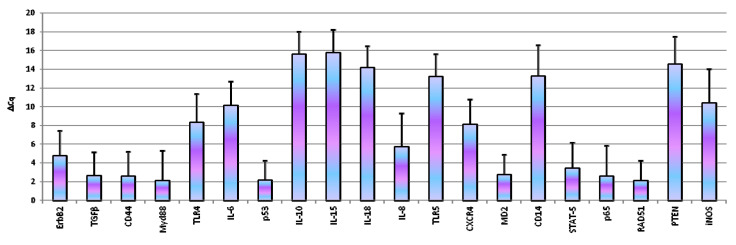
Basal gene expression in D-17 cells. Data are expressed as ΔCq ± 1 standard deviation.

For each analysed candidate reference gene, the assay was selected on the basis of the presence of a single peak in the melting curve or the lack of relevant amplification of the genomic control: in Table 1 and Table 2 the primers and the bibliographic references used for each test are reported. The assays of the reference genes that have been tested in the present study showed an efficiency from 96.7% to 111.8%, an R^2^ range from 0.987 to 1.000 and a slope from −3.000 to −3.404 (Table 1). For gene expression analysis assays ranged between 97% to 109%, and the correlation coefficient R^2^ was above 0.97 for all primers set. Based on the analysis performed with NormFinder, the best reference genes for the normalization of D-17 expression data are *RPS5* and *RPS19*. Therefore, data were normalized using *RPS5* as reference. Our results showed the expression of all the genes under study. In particular, *ErbB2, TGFβ, CD44, IL6, IL8, p65, TLR4, TP53, MD2, MyD88, STAT5, CXCR4 and RAD51* were expressed in all the samples analysed. The other genes were expressed in inconsistent manner: *iNOS* was expressed in 86.1% of analysed samples, *IL-15* in 13.9%, *IL-10* in 19.4%, *IL-18* in 41.7%, *CD14* in 50%, *PTEN* in 55.6% and *TLR5* in 80.6%. Figure 1 reported data of basal gene expression expressed as ΔCq ± 1 standard deviation.

**Figure 2:**
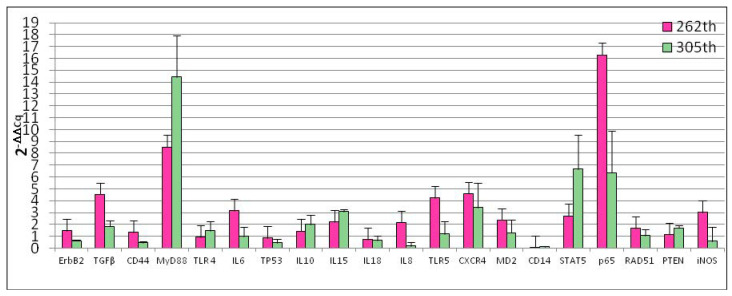
Effect of different passages on D-17 cells gene expression. Data are expressed as 2^-ΔΔCq^; values are the mean of three test replicates and ΔΔCq = ΔCq (305th or 262th passage)—ΔCq (control 255th passage). Negative samples were given a Cq 39 fictitious value. Data were checked by ANOVA for different value of significance (*p* < 0.05; *p* < 0.01; *p* < 0.001; *p* < 0.0001).

**Figure 3:**
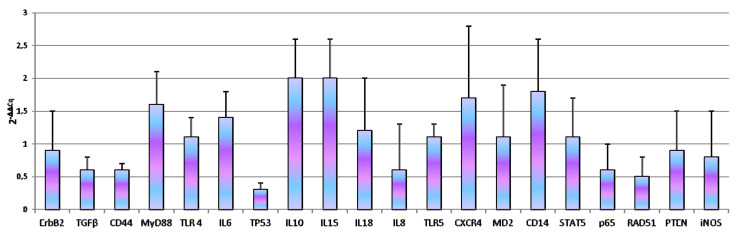
Effect of monolayer aging in D-17 cells. Data are expressed as 2^-ΔΔCq^; values are the mean of three test replicates ± 1 standard deviation and ΔΔCq = ΔCq (302nd passage at confluence plus 24 h of incubation at 37 °C)—ΔCq (control 302th passage at confluence). Negative samples were given a Cq 39 fictitious value. Data were checked by Student’s *t*-test.

Our experiments demonstrated that, the number of cellular passages significantly influenced the basal gene expression of all genes under study with the exception of *TLR4*, *iNOS*, *RAD51*, *IL10* and *IL18* (Figure 2). At 262th passages *TGFβ* (*p* = 0.0005), *MyD88* (*p* < 0.0001), *IL6* (*p* = 0.0033), *IL8* (*p* = 0.0078), *TLR5* (*p* = 0.007), *CXCR4* (*p* = 0.0054), *MD2* (*p* = 0.0151), *STAT5* (*p* = 0.0094), *p65* (*p* = 0.0033) gene expression was up-regulated while *CD14* (*p* < 0.0001) was down-regulated. Regarding 305th passages, we observed significant increase of *TGFβ* (*p* = 0.0079), *MyD88* (*p* < 0.0162), *IL15* (*p* = 0.00131) and *PTEN* (*p* = 0.00476) gene expression. *ErbB2* (*p* = 0.009), *CD44* (*p* = 0.0045), *IL8* (*p* = 0.0003) and *CD14* (*p* = 0.0006) were down-regulated. At the same time, monolayer aging (24 h of incubation) not influence gene expression (Figure 3).

### 3.2. Immuno-Cytochemicals Assay

**Figure 4:**
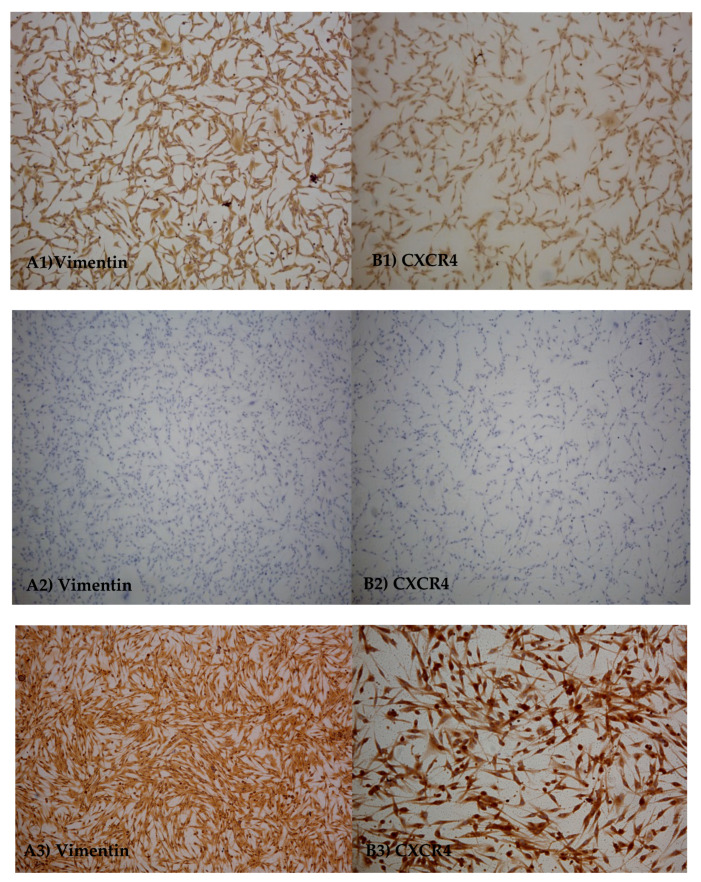
Immunocytochemical assay. Representative images: Vimentin (**A1**) and CXCR4 (**B1**) protein expression on D-17 cell line; negative control (**A2**,**B2**); positive control (**A3**,**B3**).

Immunocytochemical analysis were performed in order to assess the presence of specific markers on D-17 cells, to evaluate cell morphology and to confirm the mesenchymal origin of cell line. Cells, at 305th passages, were found to be diffusely positive for CXCR4 and VIM (Figure 4).

### 3.3. Innate Immune Response to Infective Stressor

**Figure 5:**
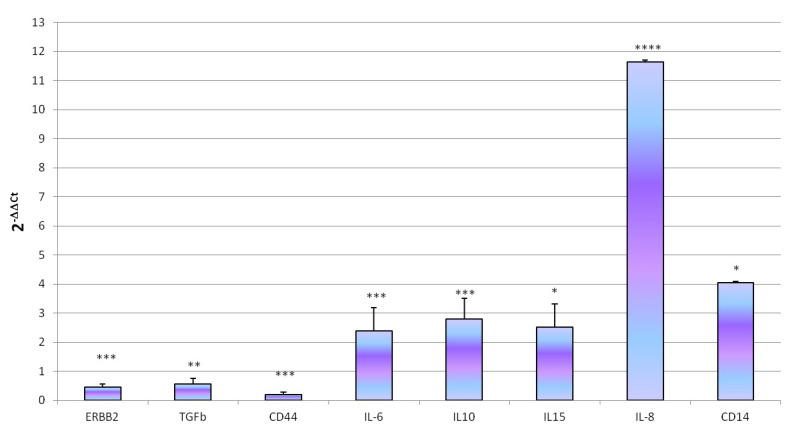
Effect of *ST* treatment on D-17 genes expression. Genes expression was measured after treatment of D-17 cells with 1 mL of bacterial suspension (MOI 100 ufc/cells) for 1 h at 37 °C in 5% CO_2_. Data are expressed as 2^-ΔΔCq^, values are the mean of three test replicates ± 1 standard deviation and ΔΔCq = ΔCq (*ST* treatment)—ΔCq (untreated control). Negative samples were given a Cq 39 fictitious value. Asterisks indicate significant differences: * *p* < 0.05, ** *p* < 0.01, *** *p* < 0.001 and **** *p* < 0.0001. (Not significant changes in genes expression were not showed).

*ST* (MOI 100 ufc/cells) showed the ability to colonize D-17 after 1 h of exposure (4.14 ± 0.14 Log10 bacteria/500,000 cells) with a penetration rate of 0.02%. *ST* exposure did not lead significant changes in cell viability (*p* < 0.05); indeed, we observed log_10_ 5.34 ± 0.19 living cells in control wells and log_10_ 5.23 ± 0.15 living cells in treated. Concerning gene expression, *ST* exposure determine modulation of gene expression characterized by a significant increase of *IL6* (*p* = 0.00028), *IL8* (*p* < 0.0001), *IL10* (*p* = 0.0041), *IL15* (*p* = 0.0136) and *CD14* (*p* = 0.027) and a decrease of *ErbB2* (*p* = 0.00021), *CD44* (*p* = 0.001) and *TGFβ* (*p* = 0.0122) genes expression (Figure 5).

### 3.4. Sequencing of the Key Gene CXCR4

**Figure 6:**
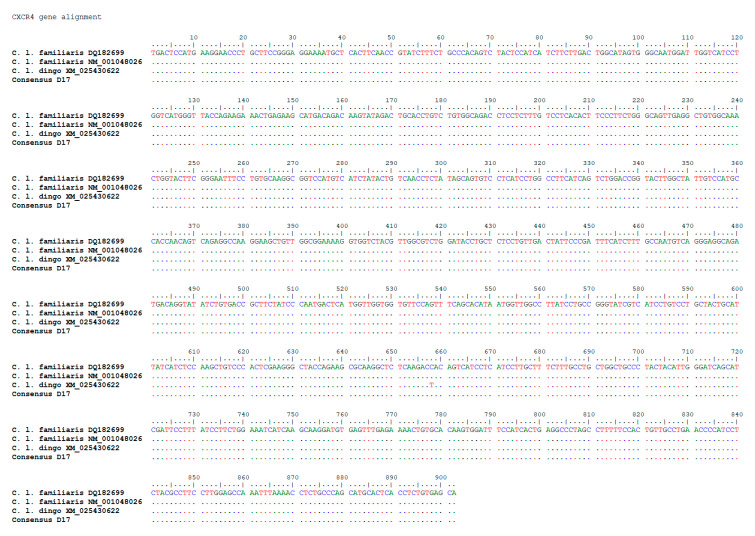
Sequencing of CXCR4 gene: alignment between the obtained sequence and reference sequences: DQ182699.1 Canis familiaris chemokine (C-X-C motif) receptor 4 (CXCR4) mRNA, complete cds; NM_001048026.1 Canis lupus familiaris C-X-C motif chemokine receptor 4 (CXCR4), mRNA; XM_025430622.1 PREDICTED: Canis lupus dingo C-X-C motif chemokine receptor 4 (CXCR4), mRNA.

Alignment was performed with BioEdit Sequence Alignment Editor version 7.2.5 using the following reference sequences from GeneBank: DQ182699.1 *Canis familiaris* chemokine (C-X-C motif) receptor 4 (CXCR4) mRNA, complete cds; NM_001048026.1 *Canis lupus familiaris* C-X-C motif chemokine receptor 4 (CXCR4), mRNA; XM_025430622.1 PREDICTED: *Canis lupus dingo* C-X-C motif chemokine receptor 4 (CXCR4), mRNA. Sequences alignment and the comparison with the sequences deposited in GeneBank enlighten both the absence of mutations on *CXCR4* gene in the serial passages of D-17 and in all other examined tissues (Figure 6).

## 4. Discussion

In humans, OSA represents severe cancer in children and young people, but the low incidence, the limited number of samples and the lack of clinical information do not allow the study of new biomarkers and of alternative therapy in humans. Canine and human OSA share common characteristics, such as prognostic factors, genetic aberrations, biological behaviors and metastatic progression, therefore the high frequency in the dog and availability of samples, make the canine OSA a good study model in comparative oncology [9,35]. Current legislation on the protection of laboratory animals has forced the scientific community to use alternative methods for experimentation, as the use of cell cultures. In this context, D-17, a cell line of canine osteosarcoma, has reached worldwide use for in vitro studies [10,36]. However, although several studies show that D-17 is optimal for these observations, this cell line has not been characterized in terms of basal gene expression, secretion and expression of proteins and in terms of their ability to respond at infectious stressors. The lack of these data does not allow optimal use of the cell line during in vitro studies, making it difficult the study of alternative molecules in the therapeutic field or the study of biological markers. In this work we tested D-17 at different passages and cells showed to maintain the same characteristics of the tissue of origin. Moreover, immunocytochemical assay showed expression of vimentin confirming the mesenchymal origin and CXCR4 in agreement with a lung metastatic OSA. In this conceptual framework, the study we carried out investigated the basal expression of important genes involved in cell cycle and in the innate immune response. In particular, D-17 had an over-expression of *IL8* [28], *TP53* [29], *ErbB2* [30] and a discontinuous expression of *PTEN* [29]. These data are in accordance with what was been highlighted in the source tumor; in fact, an altered expression of p53 is a predisposing factor for tumor development [37]. This protein acts as a tumor suppressor because it intervenes in the preservation of cell stability facilitating the repair of damaged DNA, blocking the cell cycle or inducing the apoptosis. In the case of DNA repair, the protein is degraded, and the cell cycle resumes normally. The protein p53 is therefore capable of inducing the halt of cell growth, apoptosis and cellular senescence [38]. Russell and collaborators [30] showed an increased expression of p53 in the OSA. Normally p53 has a low concentration and short half-life in healthy cells. Its presence in the OSA suggests an abnormal activity. In their work authors had showed a cytoplasmic accumulation of the protein, which might be due to an abnormal cytoplasmic translocation nuclear localization signal due to the link with an heat shock protein 70 kilodaltons (HPS70). Regarding the expression of *PTEN*, our study highlighted a discontinuous expression of this gene, one of the main tumor suppressor genes. When the gene is impaired, cells can reproduce without control [38] and the inhibition of the PI3K/AKT pathway is discontinued, resulting in the activation of the preferred glycolysis in the neoplastic cells for the ATP production compared to mitochondrial respiration [39]. Many studies have been shown the down-regulation or *PTEN* mutation/deletion in cases of OSA [29,40].

Another gene that showed an increased expression in our study was *ErbB2*, a gene that encodes a protein belonging to the family of growth factor epidermal receptors, positioned outside the cell and involved in signaling pathways that lead to cell growth and differentiation [37]. Recent studies have shown that increased expression of this gene can be associated with metastasis and fatal prognosis [31]. *IL8* is also expressed in all the analysed samples. This chemokine is known for its pro-inflammatory activity in vivo and in vitro, chemotactic on neutrophils, eosinophils, basophiles and immune cells, angiogenetic [41] and is, with VEGF, PD-ECGF, bFGF and TNFa, one of the tumor molecules derived from macrophages and associated with neo-angiogenesis. IL-8 is produced by macrophages infiltrating in and around the tumor, it can then act as angiogenic shift and be an angio-invasion index. The suppression of IL8 reduces tumor development and progression [42,43]. The data obtained in D-17 are therefore in accordance with data obtained in other studies conducted in OSA.

In our study, we evaluated the expression of *TLR4* and *TLR5*, a family of receptors, expressed by many cell types (macrophages, dendritic cells, neutrophil granulocytes, B lymphocytes, mucous epithelium cells, endothelial cells), which is tasked with recognizing typical structures of pathogens and microorganisms (bacteria, viruses, fungi), starting the innate immune response [44]. In particular, TLR5 recognizes flagellin and TLR4 the lipopolisaccaride linked to LBPs (LPS binding protein). TLR4 performs its function linking to MD2 that forms a complex with the CD14 membrane protein [45]. The expression of MD2, CD14, TLR4 and TLR5 in D-17 suggests the possibility of interacting with negative Gram bacteria [46]. Genes expressed in response to TLRs activation encode for important proteins at various levels in the immune response for example, the p65 expression associated with MyD88 activation and the expression of various chemokines (IL-6, IL-8, IL-10, IL-15, IL-18). Another molecule considered by our study and whose aberrant activity is related to various types of cancer is the STAT5 protein, expressed in the cell line of osteosarcoma. In D-17 we highlighted the basal expression of both CD44 and CXCR4; the first is a trans-membrane protein involved in the tumor microenvironment that interacts with various components of the extracellular matrix, cytokines and growth factors [32]. It is an adhesion molecule expressed on lymphocytes and leucocytes, which use it to adhere to extracellular matrix proteins during inflammatory processes and expressed in many tumors. CXCR4, on the other hand, is an exclusive receptor for cell SDF-1 or CXCL12 expressed by T cells, NK, dendritic cells and monocytes [47]. Among other activities, it also regulates the immune response. In fact, it manages the development and function of B cells [48], the transport of leukocytes and their distribution in peripheral tissues and participates in the organization of lymph nodes [49]. In addition, during bacterial infection, it averages the relocation of neutrophils between lymph nodes where the first adaptive immune response occurs [50].

This receptor has been proven to be fundamental in tumor development and progression and in particular in the pulmonary metastatization of OSA [30,34]. In our study we demonstrated that in D-17 this receptor had not mutations and was expressed; this is important finding because suggest the possibility to use D-17 to test in vitro anticancer therapies based on CXCR4 pathway.

The data we obtained in the preliminary phase confirmed that D-17 cell line has the same expression of genes as spontaneous OSA reported in literature and the maintenance of the characteristics of a metastatic pulmonary OSA. Concerning the stimulus with infectious stressors, the cell line has been able to cause an inflammatory response following exposure to *Salmonella typhimurium*. Indeed, in our study we showed up-regulation of the pro-inflammatory cytokines *IL6* and *IL8*, given in accordance with *Salmonella*’s ability to penetrate D-17; in fact it is known as this microorganism exploits inflammation to penetrate enterocytes [8]. This confirms as D-17 can interact with an infectious stressor such as *Salmonella typhimurium,* suggesting D-17 as a useful cell line for the preliminary evaluation of new bacteria-based therapeutic approaches [51].

## 5. Conclusions

In conclusion, our data highlight the maintenance by the cell line of the genes’ expression parameters typical of OSA, that is characterized by overexpression of *TP53*, *ErbB2*, *IL8* and a discontinuous expression of *PTEN*. In addition, this cell line demonstrated the ability to interact with an infectious stressor such as *ST* by suggesting the D-17 as a possible in vitro model for the preliminary evaluation of new therapeutic approaches based on the use of bacteria.

## Figures and Tables

**Table 1:** Characteristics of the primers used and the bibliographic references.

Gene	Primers	Efficiency	R^2^	Slope	References/ Designed
*RPS5*	F: TCACTGGTGAGAACCCCCT R: CCTGATTCACACGGCGTAG	106.9%	0.998	−3.167	[9]
*B2M*	F: TCCTCATCCTCCTCGCT R: TTCTCTGCTGGGTGTCG	111.8%	0.990	−3.000	[9]
*GUSB*	F: AGACGTTCCAAGTACCCC R: AGGTGTGGTGTAGAGGAGCAC	99.7%	0.998	−3.328	[9]
*GAPDH*	F: CTGGGGCTCACTTGAAAGG R: GGAGGCATTGCTGACAATC	96.7%	0.999	−3.404	Designed
*HPRT1*	F: CTGAAGAGCTACTGTAATGACCAGTC R: CTTTTCACCAGCAAGCTTGCAACC	101.6%	0.987	−3.284	Designed
*RPL13A*	F: GGGGCAGGTCCTGGTGCTCG R: CCAGGTACTTCAACTTGTTTCTGTAG	97.7%	0.999	−3.378	Designed
*RPS19*	F: CCTTCCTCAAAAAGTCTGGG R: GCTGTGGAAGCAGCTCGC	97.0%	0.999	−3.396	[10] Designed
*SDHA*	F: GGTGGCACTTCTACGACACC R: CCATAATTCTCCAGCTCTACC	103.7%	1.000	−3.237	[11] Designed

**Table 2:** Primer Set for Sybrgreen quantitative, real-time (RT)-PCR amplification of canine genes.

Gene	Primer		Product Length (bp)	Accession Number	Source
*ErbB2*	Forward	CTGAGGGCCGATATACCTTC	113	NM_001003217.2	[13]
	Reverse	TCACCTCTTGGTTGTTCAGG			
*TGFβ*	Forward	CAAGTAGACATTAACGGGTTCAGTTC	70	L34956	[14]
	Reverse	GGTCGGTTCATGCCATGAAT			
*CD44*	Forward	CAAGGCTTTCAACAGCACCC	191	NM_001197022.1	[15]
	Reverse	TACGTGTCGTACTGGGAGGT			
*IL6*	Forward	TCCAGAACAACTATGAGGGTGA	99	NM_001003300.1	[16]
	Reverse	TCCTGATTCTTTACCTTGCTCTT			
*IL10*	Forward	CGACCCAGACATCAAGAACC	100	NM_001003077.1	[17]
	Reverse	CACAGGGAAGAAATCGGTGA			
*IL15*	Forward	ACTTGCATCCAGTGCTACTT	270	AF479882.1	[18]
	Reverse	CGAGCGAGATAACACCTAAC			
*IL8*	Forward	TGATTGACAGTGGCCCACATTGTG	355	D14285.1	[15]
	Reverse	GTCCAGGCACACCTCATTTC			
*CD14*	Forward	GCCGGGCCTCAAGGTACT	60	XM_843653.4	[19]
	Reverse	TCGTGCGCAGGAAAAAGC			
*p65*	Forward	TGTAAAGAAGCGGGACCTGG	249	AB930129.1	[20]
	Reverse	AGAGTTTCGGTTCACTCGGC			
*IL18*	Forward	CTCTCCTGTAAGAACAAAACTATTTCCTT	99	NM_001003169.1	[21]
	Reverse	GAACACTTCTCTGAAAGAATATGATGTCA			
*TLR4*	Forward	GCTGGATGGTAAACCGTGGA	157	NM_001002950.2	[15]
	Reverse	AGCACAGTGGCAGGTACATC			
*TLR5*	Forward	CCAGGACCAGACGTTCAGAT	108	EU551146.1	[22]
	Reverse	GCCCAGGAAGATGGTGTCTA			
*TP53*	Forward	CGTTTGGGGTTCCTGCATTC	231	NM_001003210.1	[15]
	Reverse	CACTACTGTCAGAGCAGCGT			
*MD2*	Forward	GGGAATACGATTTTCTAAGGGACAA	91	XM_848045.3	[19]
	Reverse	CGGTAAAATTCAAACAAAAGAGCTT			
*MyD88*	Forward	GAGGAGATGGGCTTCGAGTA	159	XM_534223.5	[15]
	Reverse	GTTCCACCAACACGTCGTC			
*STAT5*	Forward	TTGACTCTCCTGACCGCAAC	181	XM_548091.5	[15]
	Reverse	TCCGTCTACTGCTTTAGCGA			
*iNOS*	Forward	AGACACACTTCACCACAAGG	284	AF07782.1	[23]
	Reverse	TGCTTGGTGGCGAAGATGAGC			
*CXCR4*	Forward	GCGTCTGGATACCTGCTCTC	163	NM_001048026.1	[15]
	Reverse	GATACCCGGCAGGATAAGGC			
*RAD51*	Forward	GGAGAAGGAAAGGCCATGTA	147	NM_001003043.1	[24]
	Reverse	GGGTCTGGTGGTCTGTGTT			
*PTEN*	Forward	GTGAAGCTGTACTTCACAA	135	NM_001003192.1	[25]
	Reverse	CTGGGTCAGAGTCAGTGGTG			

**Table 3:** Primers used for CXCR4 amplification and sequencing.

Primer		Position	Product Lenght (bp)	Accession Number	Source
*CXCR4 F*	TCT GTG GCA GAC CTC CTC TT	F 266–285 R 611–630	364	NM_001048026.1	[33]
*CXCR4 R*	TGA AAC TGG AAC ACC ACC AA				
*CXCR4 F7*	TGA CTC CAT GAA GGA ACC CTG	F 88–108 R 971–990	902	NM_001048026.1	This paper
*CXCR4 R2*	CTG CTC ACA GAG GTG AGT GC				
*CXCR4 Fow 3a*	GTC ATC CTG TCC TGC TAC TG	F 665–684 R 296–314	Sequencing 902 bp	NM_001048026.1	This Paper
*CXCR4 Rev 3b*	CAA CTG CCC AGA AGG GAA G				
